# Evaluation of Aflatoxins Occurrence and Exposure in Cereal-Based Baby Foods: An Update Review

**DOI:** 10.1007/s13668-024-00519-x

**Published:** 2024-01-29

**Authors:** Yasemin Açar, Gamze Akbulut

**Affiliations:** 1 Department of Nutrition and Dietetics, Gazi University, Ankara, Turkey; 2 Department of Nutrition and Dietetics, Istanbul Kent University, Istanbul, Turkey

**Keywords:** Aflatoxins, Breast milk, Cereal-based baby foods, Infant nutrition, Mycotoxin

## Abstract

**Purpose of Review:**

The first stages of human life, which include the fetal period, infancy, and early childhood, are the most critical for human growth and development. This is the most vulnerable phase to health challenges due to the immature immune system and rapid development. Mycotoxins such as aflatoxins, ochratoxin A, patulin, fumonisins, zearalenone, and deoxynivalenol are secondary metabolites secreted by various fungal species, primarily *Aspergillus*, *Fusarium*, *Penicillium*, and *Alternaria*. Aflatoxins are one of the major mycotoxins produced in cereals and cereal-based foods by several species of *Aspergillus*, mainly *Aspergillus flavus*. In this context, this review provides a brief overview of the occurrence, exposure, legal regulations, and health effects of aflatoxins (B1, B2, G1, G2, and M1) in cereal-based baby foods and breast milk.

**Recent Findings:**

Human aflatoxin exposure in utero and through breast milk, infant formulas, cereals, and cereal-based foods has been linked to various health consequences, including adverse birth outcomes, impaired growth and development, immune system suppression, and hepatic dysfunction. Recent evidence suggests that especially infants and children are more susceptible to aflatoxins due to their lower body weight, lowered capacity to detoxify harmful substances, more restrictive diet, immature metabolism and elimination, and faster rates of growth and development.

**Summary:**

It is essential for both food safety and infant and child health that aflatoxins in cereal and cereal-based products are precisely detected, detoxified, and managed.

## Introduction

Mycotoxins are toxic metabolites produced by certain fungi belonging to the genus *Aspergillus*, *Penicillium*, and *Fusarium*, causing health problems in humans and animals ranging from allergic responses to death [1]. Mycotoxins cause biochemical, physiological and/or pathological changes in humans, animals, plants, and other microorganisms because of exposure in various ways [2]. In the literature, the most common mycotoxins in food products are aflatoxins (AF), ochratoxin A (OTA), patulin (PAT), fumonisins (FB), zearalenone (ZEN), and deoxynivalenol (DON) [3, 4]. Among the types of mycotoxins, 20 different types of AF were identified. AFB_1_, AFB_2_, AFG_1_, and AFG_2_ are frequently reported as significant contaminants of food products [5•, 6]. Ochratoxin A is a type of mycotoxin that has carcinogenic, teratogenic, immunotoxic, and neurotoxic effects. Also, it belongs to Group 2B carcinogenic according to the IARC (International Agency for Research on Cancer) [5•, 7]. As a type of mycotoxin, PAT has been identified in various food products, including fruits and vegetables. It is mostly found in apple and apple products, in fruits including pear, apricot, peach, and grapes [8]. According to the IARC, PAT is a group 3 carcinogen, and it has a neurological, gastrointestinal, and immunological adverse effects [9, 10]. Fumonisins are mycotoxins produced by the fungi *Fusarium verticillioides* and *Fusarium proliferatum* [11]. Fumonisins have been classified by the IARC as a Group 2B possible human carcinogen [9]. Zearalenone and its derivatives are defined as estrogenic mycotoxins, and they mainly exist in moldy food and crop. The structure of ZEN is like estrogen, so it has estrogen-like effects on various organisms [12]. Deoxynivalenol, produced by *Fusarium* spp., is a mycotoxin in cereals (wheat, rye, barley, and oats) and cereal-based food products [13].

Consumption of mycotoxins may cause decreased resistance to infectious diseases and impaired immunity. Mycotoxins cause growth retardation by inhibiting protein synthesis, especially in children under the age of 5, and have adverse effects on morbidity and health [14••]. In this context, the aim of the this study was to provide an overview about the aflatoxin occurrence and worldwide exposure of this toxin in human breast milk, infant formula, and cereal-based products for infants and was evaluated regarding the published data during the past decade on aflatoxin prevalence.

### Aflatoxins

Aflatoxins are classified by IARC as group 1 carcinogens due to their toxic, carcinogenic, teratogenic, mutagenic, and immunotoxic structure. Aflatoxins are found in cereals and cereal-based products such as grains, bread, breakfast cereals, pasta products, and infant formulas [15]. After immunoaffinity column cleanup and fluorescence detection, high-performance liquid chromatography (HPLC) is commonly used to analyze aflatoxin [16]. Aflatoxins of the B and G groups are named after their blue or green fluorescence under UV light, respectively, whereas aflatoxins of the M group are named after their presence in milk and milk products [17].

In the literature, 20 different types of AF have been identified. Among them AFB_1_, AFB_2_, AFG_1_, AFG_2_, and AFM_1_ are frequently reported as the important contaminants of food products [5•]. Dietary exposure to AF in childhood occurs via breast milk and complementary infant foods [2]. A summary of the aflatoxin types, dietary sources, and chemical structures is in Table 1.

**Table 1:** Summary of the aflatoxin types and dietary sources [5•, 6]

**Aflatoxin type**	**Dietary sources**
AFB_1_	Plant-based foods Cereal and cereal-based foods
AFB_2_	Plant-based foods Cereal and cereal-based foods
AFG_1_	Plant-based foods Cereal and cereal-based foods
AFG_2_	Plant-based foods Cereal and cereal-based foods
AFM_1_	Dairy products Human breast milk

#### AFB_1_

Aflatoxin B_1_ (AFB_1_) is the most toxic aflatoxin, being categorized as Group 1 (a human carcinogen), by the IARC and is thought to be the primary cause of human liver cancer [5•]. The liver is thought to be the primary target organ for aflatoxin carcinogenesis. Thus, to reduce aflatoxin exposure, most countries have strict rules for controlling aflatoxin B_1_ in natural or formulated food products [18].

The European Union has regulated the strictest maximum levels for aflatoxin B_1_ (0.10 µg/kg) in infant and baby foods in the Commission Regulation based on the risks associated with mycotoxins in infants [19]. Although aflatoxins are carcinogenic to humans and that the four major aflatoxins may co-occur in infant cereals, no maximum levels for the sum of aflatoxins B_1_, B_2_, G_1_, and G_2_ have been established.

AFB_1_-lysine (AFB_1_-lys) levels, which have a half-life of 2–3 months, are used as a reliable biomarker to measure and evaluate AFB_1_ exposure in epidemiological studies [20]. In a study conducted by Chen et al., they examined children’s exposures to dietary AFB_1_-lys and potential impacts on growth in 114 children under 36 months of age in Tanzania. AFB_1_-lys was detected in serum samples of 72% of the children, with a mean level of 5.1 pg/mg albumin [21]. Liquid chromatography isotope dilution mass spectrometry (LC/MS) was used to determine its concentrations, as described by [22] and [23]. Urine metabolites have a half-life of a few hours or less in humans; the aflatoxin B1-albumin lysine adduct is thought to have the same half-life as albumin itself, about 3 weeks. Because exposure of aflatoxins may be evaluated over months and repeated exposures result in larger AFB_1_-Lys levels, the utilization of the aflatoxin B_1_-albumin lysine adduct, measured as aflatoxin B_1_-lysine (AFB_1_-lys), is thought to have a higher value as a biomarker [24]. In a prospective cohort study by Lauer et al., the serum concentration of AFB_1_-lys adduct was used to determine maternal aflatoxin exposure in Uganda. A relationship was found between maternal exposure to aflatoxin during pregnancy and adverse birth outcomes such as low birth weight and smaller head circumference [25]. Gichohi-Wainaina et al. assessed AFB_1_ exposure in mothers and the risk of stunting, wasting, and underweight in their children under the age of 24 months. The highest AFB_1_ contamination levels were found in maize grain samples. AFB_1_ concentration was associated with lower weight for height *z* scores and weight for age *z* scores in children [26]. In summary, high levels of AFB_1_ in mothers and children have been linked to stunting and underweight and low birth weight babies.

Nejad et al. conducted a systematic review and meta-analysis to explore the relationship of AFB_1_ on infant/children growth parameters such as wasting, underweight, stunting, and weight for-age (WAZ), height-for-age (HAZ), and weight-for-height (WHZ) *z*-scores. This is the first meta-analysis to investigate the relationship between AFB_1_ exposure and child growth parameters. The studies included in the article were conducted in countries such as Zambia, Nepal, Pakistan, Bangladesh, Tanzania, and Ethiopia. AFB_1_ exposure was found negatively associated with growth *z*-scores including WHZ and HAZ in infants/children, a possible risk factor for infant/child growth impairment [27].

#### AFM_1_

Mammals fed AFB1-contaminated diets excrete 0.3–6.3% of the main 4-hydroxylated metabolite known as aflatoxin M_1_ (AFM_1_) in their milk as a modified form of mycotoxin. The International Agency for Research on Cancer (IARC) has classified aflatoxin M_1_ as 2B (possible human carcinogen) [9]. Aflatoxin M_1_ has been classified as one of the most common chemical compounds in various dairy products such as milk, cheese, yogurt, butter, and infant formula as well as in animal tissues and human milk [28]. AFM_1_ is resistant to the high temperatures used in autoclaving and pasteurization processes; thus, it is important to reduce aflatoxin levels during milk and dairy product production, particularly during storage stages [29].

AFM_1_ can be detected in mother’s milk 12–24 h after consuming contaminated food. It rapidly decreases with time and is no longer detectable after 3 days of not consuming contaminated food [30]. Because AFM_1_ is secreted in human breast milk, AFM_1_ exposure in infants and children has been linked to Reye and Kwashiorkor’s syndromes, immunosuppression, dermal irritation, endocrine disruptions, growth retardation, underweight, and infectious diseases. Thus, investigating the presence and level of AFM_1_ in human breast milk is of particular interest [31].

The Scientific Commission of the European Community regulates a maximum limit of 0.025 µg/kg for AFM_1_ in infant formulae and follow-on formulae, including infant milk and follow-on milk [19].

In a study conduct by Kabak, it was evaluated that aflatoxin M_1_ occurs in infant formula, follow-on formula, and toddler formulae marketed in Turkey. Aflatoxin M_1_ was detected in five of the 62 samples (8%), at levels ranging from 0.016 to 0.022 µg/kg (mean level 0.018 µg/kg) but at levels below the European legislation limit of 0.025 µg/kg. It concluded that the presence of AFM_1_ in Turkish formulae does not appear to pose severe health danger to children, as none of the samples surpassed the European standard of 0.025 mg kg for AFM_1_ [32].

### Aflatoxin Exposure During the First 1000 Days of Life

Pregnancy, infancy, and early childhood are particularly vulnerable periods to environmental toxins, and any health risks associated with toxicant exposure during these critical periods of life could have long-term consequences [33]. Transplacental transport, breastfeeding, and complementary feeding are important routes of dietary exposure to these contaminants during gestation and early postnatal life [34]. Aflatoxin exposure in utero may contribute to negative pregnancy outcomes, such as impaired fetal growth, premature delivery, and pregnancy losses. In addition, maternal exposure to aflatoxins during pregnancy can result in adverse birth outcomes, such as low birth weight, small-for-gestational-age, preterm birth, and poor growth that lasts into infancy and early childhood [33, 35, 36]. In a systematic review conducted by Alvito et al., they evaluated 17 epidemiological studies and their relationship between adverse pregnancy outcomes and maternal mycotoxin exposure. They found an adverse effect of maternal aflatoxin exposure on fetal growth, a decreased birth weight, and an increased risk of low birth weight among exposed newborn infants [37]. In another prospective cohort study conducted by Tesfamariam et al., the association between chronic aflatoxin exposure during pregnancy and fetal growth trajectories was examined. In this study, aflatoxin was found in 86.6% of maternal blood samples (*n*, 492), and the aflatoxin-exposed group showed a significantly lower change in fetal weight-for-gestational-age centile change over time than the unexposed group [38].

Infants and children are the most vulnerable to aflatoxins because of their lower body weight, decreased ability to detoxify hazardous agents, more restricted diet, immature metabolism and elimination, and faster growth and development rates [39, 40]. Furthermore, some nutritional factors influence aflatoxin toxicity. Children who are protein deficient, for example, are more sensitive to aflatoxins [5•]. Aflatoxin exposure is associated with childhood kwashiorkor and marasmus (malnutrition-related childhood disease). Protein deficiency is a major cause of kwashiorkor and marasmus. Children with kwashiorkor or marasmus have higher levels of aflatoxins or their metabolites in their blood and urine. In these malnutrition-related diseases, liver function has reduced, and aflatoxin metabolism has altered. For this reason, children with kwashiorkor or marasmus are more vulnerable to the hazards and toxicity of aflatoxin in their nutrition [41, 42].

**Fig. 1:**
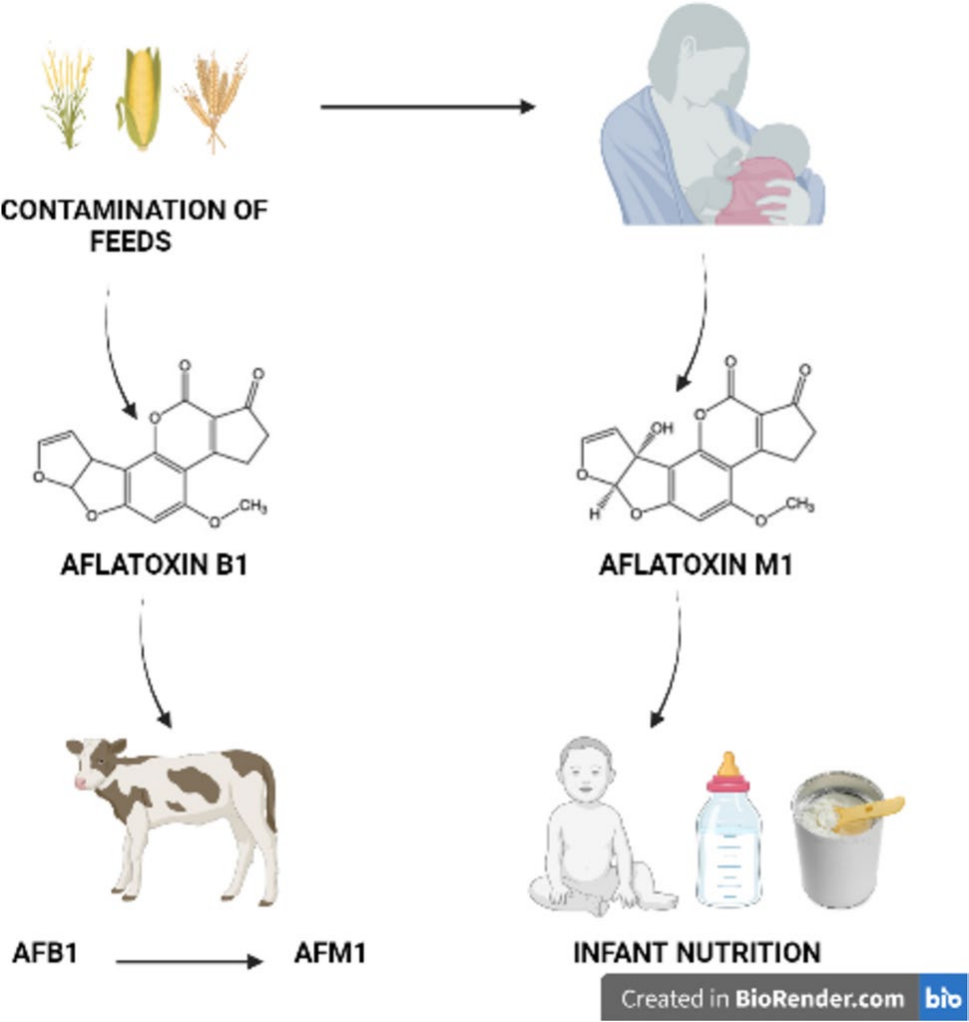
The mechanism of aflatoxin and infant nutrition

Cereal-based baby foods, which are frequently the first solid meal used in infant feeding, gradually replace breast milk during the first several months of life, mainly after 6 months of their birth [43]. Cereals (wheat, corn, oats, rice, barley, malt, soy, and rye), honey, sugar, dried fruits, and cocoa are among the ingredients in these products [44]. Cereal-based foods are sources of energy, starch, fiber, protein, high amounts of vitamins, minerals, and bioactive compounds [45]. It contains indigestible carbohydrates that are central to increasing the intestinal microbiota population. When infants are weaned, a cereal-based feeding increases the fermentation activity of the gut microbiota population [18]. Because these foods have a mild taste and semi-solid texture, they are the good choice for babies who are transitioning from breast milk to solid foods at the start of complementary feeding [46]. Despite all the benefits mentioned, the presence and exposure to aflatoxins because of the consumption of these products is unavoidable because of different grains that are used as ingredients in most baby foods for infants and children, and the likelihood of multiple mycotoxins increases [47]. To protect vulnerable populations, surveillance studies to determine the extent of contamination with the five aflatoxins in foods intended for infants and children are required [48]. The mechanism of aflatoxin and infant nutrition is summarized in Fig. 1. Studies that examine the relationship between aflatoxin exposure and child growth and aflatoxin occurrence of infant foods in different countries are summarized in Tables 2 and 3.

**Table 2:** Summary of the studies on the association between aflatoxin exposure and child growth parameters

**Country**	**Study design**	**Study population**	**Aflatoxin type**	**Analysis technique**	**Detection rat**e	**Outcome**	**Finding**	**Reference**
Nepal	Longitudinal birth cohort	1675 mother and infants (from pregnancy to 2 years of age follow-up)	AFB_1_-lysine AFM_1_	HPLC	1.54 pg/mg 0.04–315.99 ng/L (%94.1)	LAZ, WAZ, WLZ z-scores, length, knee-heel length and stunting	AFB_1_-lysine adduct concentrations were significantly associated with changes in LAZ, length, knee-heel length and WAZ z-scores. Serum aflatoxin concentrations were associated with stunting	[49]
Zambia	Cross-sectional	400 mothers with children aged 6–24 months	AFB_1_-lys	LC–MS/MS	NR	HAZ, WAZ and WHZ z-scores, stunting, underweight, and wasting	AFB_1_-lys level were found to be significantly associated with child stunting	[24]
Tanzania	RCT	Intervention group *n* = 150, control group *n* = 150	Total AFs (AFB_1_, AFB_2_, AFG_1_ and AFG_2_)	HPLC	NR	WAZ *z*-score, dietary assessment	Mean concentration of AFs was significantly lower in the intervention than control group. Mean WAZ *z*-score difference between the groups was 0.57 (*p* < 0.05)	[50]
Kenya	RCT	Intervention group *n* = 489, control group *n* = 392 (mother and infants)	AFB_1_-lys	HPLC	Intervention group: 2.69 pg/mL Control group: 2.74 pg/ mL	LAZ *z*-score, stunting and child serum AFB_1_-lysine adduct	The intervention significantly reduced endline in serum AFB_1_-lysine adduct levels but had no effect on endline LAZ or stunting	[51]
Nepal	Longitudinal cohort	85 children (15, 24, and 36 months follow-up)	AFB_1_-lys	UPLC	3.62 pg/mg	LAZ, WAZ and WLZ z-scores	The chronic AF exposure was not significantly associated with anthropometric *z*-scores	[52]
Pakistan	Cross-sectional	150 children aged 3–5 years	AFM_1_	ELISA	0.09 ± 0.32 ng/ml	HAZ, WAZ, and WHZ *z*-scores	A non-significant correlation was recorded between urinary AFM_1_ levels and the evaluated anthropometric indices	[53]
Tanzania	Longitudinal cohort	143 infants (1st, 3rd and 5th months follow-up)	AFM_1_ (breast milk)	HPLC	AFM_1_ levels ranging from 0.01 to 0.55 ng/ml	WAZ, HAZ and WHZ z-scores	A significant association was observed between AFM1 exposure levels and WAZ or HAZ z-scores	[2]

*AF* aflatoxin, *HAZ* height-for-age, *HPLC* high-performance liquid chromatography, *LAZ* length-for-age, *LC–MS/MS* liquid chromatography-mass spectrometry tandem mass spectrometry, *NR* not reported, *UPLC* ultra performance liquid chromatography, *RCT* randomized controlled trial, *WAZ* weight-for-age, *WHZ* weight-for-height, *WLZ* weight-for-length

**Table 3:** Occurrence of aflatoxins in infant foods in different countries

**Type of aflatoxin**	**Dietary source**	**Country**	**Method of detection**	**Sample size**	**Positive samples (%)**	**Mean** **concentration of AF**	**Reference**
AFB_1_	Cereal-based baby foods	Lebanese	ELISA	42	ND	ND	[54]
AFB_1_	Cereal-based baby foods	Iran	HPLC	48	33 (68.7)	2.6 ± 4.0 µg/kg	[55]
AFB1	Cereal-based baby foods	Kosovo	ELISA and LC-MS/MS	103	64 (62.1)	Ranged from 0.008 to 0.116 μg/kg	[56]
AFM1	Baby milk			40	6 (15)	Ranged from 0.008 to 0.123 μg/kg	
AFB_1_	Cereal-based baby foods	Turkey	LC-MS/MS	85	11 (12.9)	0.03 ± 0.01 (Mean ± SD)	[57]
AFB2					3 (3.5)	0.05 ± 0.01 (Mean ± SD)	
AFG1					ND	ND	
AFG2					ND	ND	
AFB_1_	Cereal-based baby foods	Spain	HPLC	60	11 (18.3)	0.03 ± 0.05 (Mean ± SD)	[48]
AFB2					1 (1.7)	0.01 ± 0.02 (Mean ± SD)	
AFG1					6 (10)	0.02 ± 0.04 (Mean ± SD)	
AFG2					1 (1.7)	0.01 ± 0.01 (Mean ± SD)	
AFM_1_	Baby formula	Lebanese	ELISA	84	8 (9.5)	5.72 ± 0.014 ng/L	[54]
AFM_1_	Breast milk	Mexico	ELISA	123	123 (100)	17.04 ng/L	[31]
AFM1	Breast milk	Turkey	HPLC	100	53 (53)	6.36 ng/L	[58]
AFM_1_	Breast milk (BM) and infant powdered milk (IPM)	Brazil	HPLC	94 (BM) 16 (IPM)	5 (5.3) 7 (43.8)	0.018 ± 0.005 (BM) 0.024 ± 0.01 (IPM)	[59]
AFM_1_	Breast milk	Iran	ELISA	85	85 (100)	5.91 ng/L	[60]
AFM_1_	Baby formula	Turkey	HPLC	62	5 (8)	0.018 μg/kg	[32]

*AF* aflatoxin, *ND* not determined, *BM* breast milk, *IPM* infant powdered milk

### In Vitro Bioaccessibility of Aflatoxins

Bioaccessibility, refers to the portion of a food contaminant that is released into the gastrointestinal tract, whereas bioavailability refers to the portion of an ingested food contaminant that enters the systemic circulation and can negatively impact health [61]. In vivo bioavailability studies are more difficult in terms of time, ethics, and cost than in vitro methods [61]. The bioavailability of nutrients is generally studied using in vitro systems based on the compartments of the gastrointestinal tract [62]. In vitro bioavailability analyses were performed in simulated mouth, stomach, and small intestine models [63]. The food product, level of contamination, and method of contamination all affect mycotoxin bioaccessibility. By simulating the digestion in vitro, mycotoxins’ bioaccessibility and bioavailability are assessed [64, 65]. Many studies have used different in vitro digestion models to determine the bioaccessibility or absorption of mycotoxins, avoiding the use of more complex cell cultures and the use of animals in in vivo experiments [66].

### Legal Regulations

The World Health Organization (WHO) has identified AFs as a global food safety concern [67]. Because of their toxic, carcinogenic, mutagenic, teratogenic, and immunotoxic properties, AFs were classified as group 1 carcinogens by the IARC [9]. Countries have implemented strict regulations to prevent AF contamination in food and feed due to serious health complications in humans and animals. The maximum permissible limit for total AFs according to the European Commission Regulation is present in Table 4 [19].

**Table 4:** Maximum limits of AFB_1_ and AFM_1_ in cereal-based products and baby formulas according to the European Commission

**Legal regulations**	**Infant products**	**Type of aflatoxin**	**Maximum limits**
European Commission 1881/2006/EC	Cereal-based and infant foods	AFB_1_	0.1 µg/kg
	Infant formula and follow-on formula	AFM_1_	0.025 µg/kg

## Conclusions and Future Perspectives

Humans can be exposed to AFs during the early stages of life, including in utero exposure, breast milk, infant formula milk, and cereal-based infant foods used up to the age of 2. Infants and children are among the vulnerable groups in the population in terms of their physiology and nutrition. The presence of AFs in infant nutrition is of high importance. Aflatoxin contamination is inevitable due to the presence of many components such as cereals (wheat, corn, oats, rice, barley, malt, soy, and rye, milk powder, and fruits in baby products). In the studies examined recently, it is seen that the presence of aflatoxins is quite common in cereal-based baby products, milk, and dairy products. The presence of AFB_1_ in cereal-based baby products and AFM_1_ in milk and dairy products can cause negative health consequences for infant and child health. Due to their thermostable nature, most mycotoxins are resistant to food processing techniques. In this context, by periodically testing baby foods for aflatoxins, safe food will be provided for infant and child nutrition.

Breast milk and continuity of breastfeeding are very important for infant nutrition. The American Academy of Pediatrics (AAP) and WHO recommend exclusive breastfeeding for approximately 6 months after birth and continued breastfeeding with complementary foods for at least 2 years. Since the mother’s nutrition will affect the composition of breast milk, it is important for public health and mycotoxin contamination to inform mothers about the consumption of foods containing mycotoxins, especially during pregnancy and breastfeeding, and to carry out the necessary legal regulations and inspections on food safety.

There are differences between countries regarding the presence of mycotoxins in breast milk, formula milk, and/or cereal-based baby foods. Epidemiological studies in the literature examining the effects of mycotoxin contamination on growth parameters in infants and children were generally conducted in countries such as Gambia, Nigeria, Kenya, Pakistan, Tanzania, Ethiopia, and Nepal. In countries with low income, ensuring food safety and carrying out legal regulations to prevent mycotoxin contamination are important to protect the health of mothers, infants, and children.

In this context, to prevent AF contamination in cereal-based baby foods, milk, and dairy products the limit of legal regulations should not be exceeded, and AF levels should be strictly monitored in high-risk areas and in commercially sold products.
